# An Aggressive Form of Langerhan Cell Histiocytosis in an Adult: Therapeutic Challenges

**DOI:** 10.1155/2017/9064326

**Published:** 2017-12-03

**Authors:** Karan Seegobin, Satish Maharaj, Cherisse Baldeo, Amal Shukri, Fauzia N. Rana

**Affiliations:** ^1^Department of Internal Medicine, University of Florida College of Medicine-Jacksonville, Jacksonville, FL, USA; ^2^Department of Pathology, University of Florida College of Medicine-Jacksonville, Jacksonville, FL, USA; ^3^Department of Haematology and Oncology, University of Florida College of Medicine-Jacksonville, Jacksonville, FL, USA

## Abstract

Langerhans cell histiocytosis (LCH) is rare in adults. Regular follow-up is mandatory due to reoccurrence. A 35-year-old male with an incidental left iliac bone lesion was diagnosed with LCH. He later became symptomatic with hip pain and spread of the disease. Despite excision of the symptomatic iliac lesion, he had progression while on cytarabine and nivolumab, evidenced by increased bone pain and involvement of other bones on imaging. He underwent excision of the jaw lesion followed by vinblastine; he was pain free and had stable disease on PET imaging after 3 months. LCH is an uncommon neoplasia. Treatment is reserved for symptomatic patients while asymptomatic patients are observed. Follow-up is imperative due to the risk of reoccurrence. Despite surgical treatment together with one of the front-line agents for refractory disease, in this case cytarabine, he still had progression of the disease. Furthermore, the trial of nivolumab was of no benefit. This case highlights good response to vinblastine which is previously reported to have good success. No trials are published, and the optimal strategy has yet to be defined. LCH with multiple bony involvement can be aggressive and therapeutically challenging.

## 1. Background

Langerhans cell histiocytosis is rare and more commonly reported in children [1]. Due to its rare nature, therapeutic management is controversial, especially in adults [2]. Reoccurrence or spread of the disease is possible; therefore, regular follow-up is important in these patients [2]. This case highlights an aggressive form of LCH that responded to vinblastine after failing cytarabine and nivolumab. There exists need for a consensus on the treatment of this rare disease.

## 2. Case

**Figure 1:**
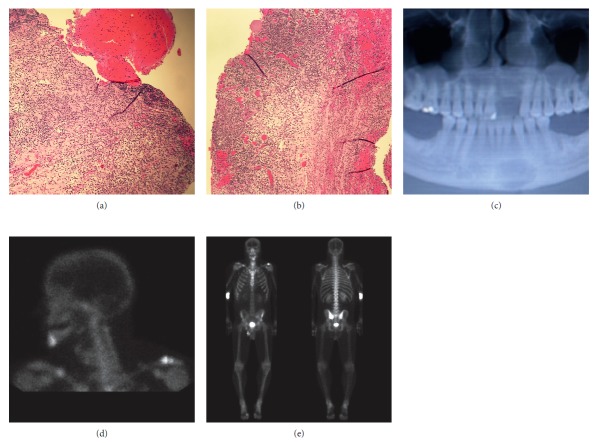
Histology of left iliac bone biopsy (a-b), X-ray of the mandible with lytic lesion (c), and bone scintigraphy images (d-e).

**Figure 2:**
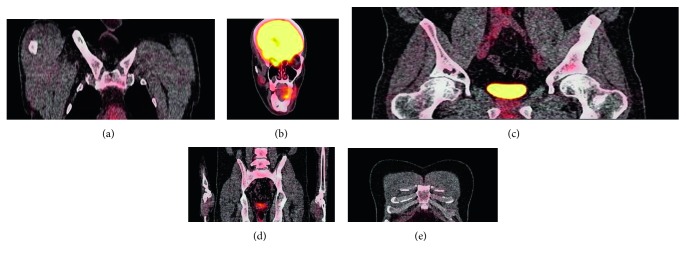
PET CT imaging without metabolic activity in stable lesions and no new lesions. (a) Right clavicle, (b) mandible region, (c) hip and pelvis, (d) pelvis and sacroiliac joint, and (e) sternum.

A 35-year-old male initially presented for evaluation of a left iliac crest lytic lesion diagnosed incidentally on CT imaging. He was asymptomatic without pain or swelling. His bone scintigraphy showed minimal uptake at the left iliac lesion without other abnormalities. CT-guided needle biopsy was done, and histology (Figures 1(a) and 1(b)) and immunohistochemical analysis were immunoreactive for S100 and CD1a, consistent with Langerhans cell histiocytosis. Watchful waiting was pursued, and he was followed up four months later which showed no change in the size of the lesion. He was then advised follow-up in one year. The patient did not keep subsequent follow-up until two years after when he started experiencing pain at the left pelvis. His laboratory workup showed sodium 135 mmol/L (135–145), potassium 4.0 mmol/L (3.3–5.1), creatine 0.81 mg/dL (0.8–1.2), calcium 9.8 mg/Dl (8–10.6), and phosphorus 4.3 mg/dL (2.7–4.5); his white cell count was 10 thou/cu mm (4–10), haemoglobin 15.2 g/dL (13–16.5), and platelet 349 (150–450 thou/cu mm. Bone scintigraphy scan showed increased uptake at the left iliac, left proximal femur, and left mandible. He subsequently underwent surgical excision of the left iliac lesion, followed with cytarabine for 6 months. While on treatment with cytarabine, he experienced jaw pain with enlargement of the lesion (Figure 1(c)). Cytarabine was stopped, and trial of nivolumab was started. However, despite nivolumab, he still had progression of the disease. His jaw pain persisted, and bone scintigraphy (Figures 1(d) and 1(e)) showed new lesions in the distal right humerus, sternum, and right midclavicle. Nivolumab was stopped, and he underwent surgical excision of the mandibular lesion followed with 6 cycles of vinblastine. The bone pain resolved, and three months later, his bone lesions remained stable without metabolic activity on follow-up PET CT imaging (Figure 2) without new lesions. He resumed work and was able to go to the gym and perform heavy weight lifting. Further treatment was planned; however, he preferred to hold off on further chemotherapy.

## 3. Discussion

Langerhans cell histiocytosis (LCH) is a rare inflammatory myeloid neoplasia occurring in both children and adults [1]. Cases are frequently reported under the age of 15 years [1]. Oral manifestations could be the first sign of disease [1]; however, it initially presented as an incidental pelvic lesion in this case. Treatment is reserved for symptomatic patients, while asymptomatic patients are kept under observation because some lesions resolve spontaneously [2]. It is imperative that follow-up studies be done as the disease can reoccur [2]. Recurrences after initial treatment occur 12–18 months after [2]. In our patient despite treatment with one of the front-line agents for refractory disease, in this case cytarabine [3], he still had spread of the disease. Furthermore, the trial of nivolumab was of no benefit. Local treatment with excision and systemic chemotherapy has been reported to be highly successful in treating this disease [4]. However, in our case the patient still experienced bone pain after excision of the mandibular and iliac lesion. The main aim is to resolve the signs and symptoms of the disease [2]. Despite initial surgery and chemotherapy in our patient, there was persistence and spread of the disease highlighting its aggressive nature.

Vinblastine has been reported among others to be successful in patients with aggressive disease [5]. After the initial treatment with vinblastine, he moved from having progressive active disease to regressive active disease based on the histiocyte society guidelines [6]. In a retrospective multicentre study, vinblastine was shown to have good response in adults as a first line treatment; however, many patients experienced reactivation in long-term follow-up [7]. Additionally, individual case reports have showed good success with vinblastine and steroids [7]. Unlike these reports which treated patients with steroids and vinblastine, our patient was treated solely with vinblastine and had excellent symptomatic improvement. No systematic series of treatments for adults with LCH have been published, and the optimal strategy has yet to be defined [5, 7]. In this case, the patient remained asymptomatic with stable PET imaging three months after 6 cycles of vinblastine. While our case and other reports demonstrate success with vinblastine, there remains need for a consensus on the treatment of aggressive Langerhans cell histiocytosis in adults.

## 4. Conclusion

This case teaches us that LCH can present as an aggressive form in adults which can be therapeutically challenging. Regular long-term follow-up is essential as multiple bony involvement can occur. Vinblastine may be beneficial in adults with aggressive disease; however, further trials are needed to help support this.
